# Extended-spectrum beta-lactamase-producing *Escherichia coli* from pork in Muang district, Chiang Mai Province, Thailand

**DOI:** 10.14202/vetworld.2022.2903-2909

**Published:** 2022-12-21

**Authors:** Wirunchana Srichumporn, Warangkhana Chaisowwong, Montira Intanon, Kannika Na-Lampang

**Affiliations:** 1 Faculty of Veterinary Medicine, Chiang Mai University, Chiang Mai, 50100, Thailand; 2 Department of Veterinary Bioscience and Public Health, Faculty of Veterinary Medicine, Chiang Mai University, Chiang Mai, 50100, Thailand; 3 Center of Excellence in Veterinary Public Health, Faculty of Veterinary Medicine, Chiang Mai University, Chiang Mai, 50100, Thailand

**Keywords:** antimicrobial resistance, extended-spectrum beta-lactamases-producing *Escherichia coli*, pork, Thailand

## Abstract

**Background and Aim:**

Contaminated pork is one of the transmission routes for pathogens. Extended-spectrum beta-lactamases (ESBL)-producing *Escherichia coli* is one of the critical threats to global public health. This study aimed to examine pork from different types of markets in Muang district, Chiangmai Province, Thailand, for a proportion of ESBL-producing *E. coli*, antibiotic resistance of ESBL-producing *E. coli* and ESBL-producing *E. coli* genotypes.

**Materials and Methods:**

Samples were collected from different market types; fresh markets, pork stores, and supermarkets, enriched and inoculated on selective media. Extended-spectrum beta-lactamases-producing *E. coli* was identified using double-disk diffusion method according to Clinical and Laboratory Standards Institute 2016. Antibiotic susceptibility test was performed through VITEK^®^ System and ESBL-encoding genes were detected using a multiplex polymerase chain reaction.

**Results:**

About 69% of the samples were positive to ESBL-producing *E. coli* and showed high rates of resistance for ampicillin (100%), piperacillin (100%), cefalexin (100%), cefpodoxime (100%), cefovecin (100%) and ceftiofur (100%), gentamycin (89.86%), and tetracycline (TE) (84.06%). All isolates were multiple drug resistant; resistance patterns of beta-lactams, aminoglycosides, TEs, nitrobenzene derivatives, and sulfonamide groups were observed. The ESBL-producing *E. coli-*positive isolates carried *bla*CTX-M groups (100%), *bla*TEM (98.55%), and *bla*SHV (1.45%). None of *bla*OXA was found in this study.

**Conclusion:**

Extended-spectrum beta-lactamases-producing *E. coli* was found in various types of markets; all isolates were detected as multidrug-resistant. The dissemination of such strains can conceivably cause concerning public health, implying that supervised antimicrobial use in pork production and sanitary food preparation is recommended.

## Introduction

Pork is one of the most consumed animal products in Thailand and worldwide. Department of Livestock Development (DLD) and National Institute of Development Administration of Thailand estimated that pigs slaughtered for consumption in 2020 to be over 6 million [1]. Consumers commonly come in contact with extended-spectrum beta-lactamases (ESBL)-producing *Escherichia coli* through contaminated meat, especially pork, from substandard production. The previous study in Chiangmai showed more than 40% of *E. coli* in pork [2] and swine production chains in the northern part of Thailand, including pigs, farmers, and environment represented more than 50% of ESBL-producing *E. coli* [3, 4]. In the northern part of Thailand, including Chiang Mai, a traditional dish called “Laab” which is spicy minced raw pork salad is common in the region. The name “Laab” is a homophone of a Thai word which means luck, making it relatively preferable. In addition, local people prefer raw meat than cooked one [5]. Extended-spectrum beta-lactamases-producing *E. coli* can be transmitted to humans through the consumption of contaminated food [6, 7].

Antimicrobial resistance (AMR) is one of the ten significant threats to global health [8]. Antibiotics were discovered in the early 20^th^ century as a potential treatment for infections. However, widespread misuse and overuse of antibiotics have increased antibiotic-resistant pathogens. Consequently, infections with AMR become common and resistant to multiple types of antibiotics [9]. Antimicrobial resistance causes inefficiency of antibiotics, increases morbidity and mortality, and impacts the world’s economy [10]. Around 500,000 human deaths each year are associated with AMR. Death caused by AMR is estimated to be approximately 10 million by 2050 [11]. Extended-spectrum beta-lactamases-producing *E. coli* is resistant to various classes of antibiotics, including beta-lactams (except carbapenems and cephamycins), aminoglycosides, and fluoroquinolones, mainly due to the production of *bla*CTX-M, *bla*TEM, *bla*SHV, and *bla*OXA genes. These pathogens have evolved and increased the transmission of antibiotic-resistant genes among farmers, animals, and animal products in Thailand [12]. Such strains are also found in meat in the Netherlands and United Kingdom [13, 14], vegetables in South Korea [15], and ready-to-eat foods in China [16].

Although pork is a common animal product in Thailand, there are relatively less data concerning ESBL-producing *E. coli* in pork from markets. Consequently, the objective of this study was to investigate the proportion of ESBL-producing *E. coli*, antibiotic resistance of ESBL-producing *E. coli*, and ESBL-producing *E. coli* genotypes. The study aims to raise awareness of antibiotic use in pork production and the dissemination of ESBL-producing *E. coli* throughout the food chain.

## Materials and Methods

### Ethical approval

Ethical approval was not required for this study.

### Study period and location

This study was conducted from January 2019 to March 2020 at the biosecurity level-2 facilities of Virology and Molecular Diagnostic Laboratory, Faculty of Veterinary Medicine, Chiang Mai University, Chiang Mai, Thailand.

### Sample collection

One hundred samples were collected from three types of markets in Muang, Chiang Mai, Thailand; 15 fresh markets (36 samples), ten pork stores (21 samples), and six supermarkets (43 samples). To illustrate, fresh markets supply fresh meat in community areas; pork stores particularly sell fresh meat from their own farms and supermarkets sell fresh meat from the suppliers with whom they signed contracts. A total of 100 samples were collected by 1–3 samples per shop, stored in a cooler box (4°C), and transported to the laboratory for bacteria isolation within 24 h. The sample collection was conducted from January 2019 to July 2019.

### Bacterial isolation and identification

25 g of pork samples was mixed with 225 mL of Luria-Bertani broth (HiMedia, India) in a sterile stomacher bag at 37°C for 24 h. Then, a loopful of suspension was streaked onto MacConkey agar (Merck, Germany) supplemented with cefotaxime 1 mg/L and incubated at 37°C for 24 h [17]. Presumptive ESBL-producing *E. coli* colonies were selected from each plate and screened using biochemical tests [18]. After that, isolation from each sample was picked to be processed through phenotypic and genotypic tests for ESBL-producing *E. coli* and antimicrobial susceptibility test.

### Phenotypic ESBL-producing *E. coli* testing

Double-disk diffusion method was conducted for ESBL detection. Mueller Hinton agar (HiMedia) was swabbed using a suspension of a pure culture (0.5 McFarland Standard). Cefotaxime (30 μg) disks (Oxoid, Germany), cefotaxime with clavulanic acid (30 μg/10 μg) disks (HiMedia), ceftazidime (30 μg) disks (Oxoid), and ceftazidime with clavulanic acid (30 μg/10 μg) disks (HiMedia) were loaded on the agar. Each antimicrobial agent combined with clavulanate was tested in comparison with the same agent without clavulanate. The agent with clavulanate combination with the increase in zone diameter measurement of more than 5 mm compared to the one without clavulanate is confirmed ESBL phenotypic [19].

### Antimicrobial susceptibility test

The minimum inhibitory concentration of antibiotic test was carried out using VITEK^®^ 2 compact (bioMérieux, Craponne, France) with AST-GN 65 cards. Drugs tested for antimicrobial susceptibility were ampicillin (AM), amoxicillin/clavulanic acid (AMC), piperacillin (PIP), cefalexin (CN), cefpodoxime (CPD), cefovecin (CFO), ceftiofur (CFT), imipenem (IPM), amikacin (AN), gentamicin (GM), tobramycin (TM), enrofloxacin (ENR), marbofloxacin (MRB), tetracycline (TE), nitrofurantoin (FT), chloramphenicol (C), and trimethoprim/sulfamethoxazole (SXT).

### Genotypic ESBL-producing *E. coli* testing and polymerase chain reaction (PCR) assay for gene detection

All positive phenotypic ESBLs were tested using PCR amplification to identify the presence of *bla*TEM, *bla*SHV, *bla*OXA, *bla*CTX-M, *bla*CTX-M-1, *bla*CTX-M-2, *bla*CTX-M-8, *bla*CTX-M-9, and *bla*CTX-M-25. DNA was extracted using NucleoSpin^®^ (Düren, Germany) according to the manufacturer’s instructions. The PCR reactions were amplified with specific primers and optimized annealing temperature for each primer (Table-1) [20, 21] through KOD One™ PCR Master Mix Blue (Toyobo, Japan) containing KOD DNA polymerase.

**Table-1:** Primers used for detecting genotypes of ESBL-producing *Escherichia coli*.

Target genes	Primers	Sequence	Annealing temp	Size (bp)	Reference
*bla*CTX-M	CTX-M-f	ATG TGC AGY ACC AGT AAR GTK ATG GC TGG	62	593	[20]
	CTX-M-r	GTR AAR TAR GTS ACC AGA AYC AGC GG			
*bla*TEM	TEM-f	CGC CGC ATA CAC TAT TCT CAG AAT GA ACG	62	445	[20]
	TEM-r	CTC ACC GGC TCC AGA TTT AT			
*bla*OXA	OXA-f	ACA CAA TAC ATA TCA ACT TCG C	65	813	[20]
	OXA-r	AGT GTG TTT AGA ATG GTG ATC			
*bla*SHV	SHV-f	CTT TAT CGG CCC TCA CTC AA	64	237	[20]
	SHV-r	AGG TGC TCA TCA TGG GAA AG			
*bla*CTX-M-1	Mul5CTXM1-f	AAA AAT CAC TGC GCC AGT TC	54	415	[21]
	Mul5CTXM1-r	AGC TTA TTC ATC GCC ACG TT			
*bla*CTX-M-2	Mul5CTXM2-f	CGA CGC TAC CCC TGC TAT T	54	552	[21]
	Mul5CTXM2-r	CCA GCG TCA GAT TTT TCA GG			
*bla*CTX-M-8	Mul5CTXM8-f	TCG CGT TAA GCG GAT GAT GC	54	666	[21]
	Mul5CTXM8-r	AAC CCA CGA TGT GGG TAG C			
*bla*CTX-M-9	Mul5CTXM9-f	CAA AGA GAG TGC AAC GGA TG	54	205	[21]
	Mul5CTXM9-r	ATT GGA AAG CGT TCA TCA CC			
*bla*CTX-M-25	Mul5CTXM25-f	GCA CGA TGA CAT TCG GG	54	327	[21]
	Mul5CTXM25-r	AAC CCA CGA TGT GGG TAG C			

ESBL=Extended-spectrum beta-lactamases

### Statistical analysis

The data were analyzed using R software version 4.0.3 (R development Core Team, R Foundation for Statistical Computing, Vienna, Austria). The unit of analysis was ESBL-producing *E. coli* isolations. Descriptive statistics were used to explain the proportions of positive ESBL-producing *E. coli*, the patterns of antimicrobial susceptibility, and ESBL-producing *E. coli* genes. Chi-square statistics and Fisher’s exact test were conducted to compare the proportions of positive ESBL-producing *E. coli* among three particular market types and represented positive ESBL-producing *E. coli* genes which were statistically significant test results (p ≤ 0.05).

## Results

### Identification of ESBL-producing *E. coli*

About 69% (69/100) samples were tested positive for ESBL-producing *E. coli* (69/100). The percentage of positive ESBL-producing *E. coli* was highest in fresh markets at 97.22% (35/36), followed by pork stores at 80.95% (17/21) and supermarkets at 39.53% (17/43). The proportions of positive ESBL-producing *E. coli* among the three market types showed statistically significant differences (p < 0.01).

### Antimicrobial resistance profiles of ESBL-producing *E. coli*

**Figure-1:**
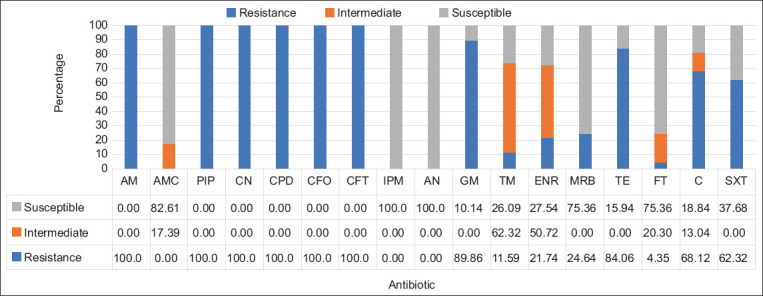
Antibiotic resistance rate of extended-spectrum beta-lactamases-producing *Escherichia coli*.

The results of the antimicrobial susceptibility test on 17 antibiotics suggested that 100% of positive ESBL-producing *E. coli* were resistant to AM, PIP, CN, CPD, CFO, and CFT, followed by GM (89.86%), and TE (84.06%). However, ESBL-producing *E. coli* were susceptible to carbapenem (IPM) (100%) and AN (100%), followed by AMC (82.16%), MRB (75.36%), and FT (75.36%) (Figure-1).

**Figure-2:**
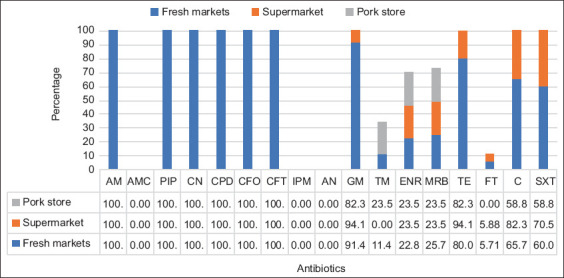
Antibiotic resistance rates of extended-spectrum beta-lactamases-producing *Escherichia coli* by market types.

All isolates were multiple drug resistant, showing resistance to at least six antibiotics. Patterns of antibiotic resistance were significantly detected in beta-lactams, aminoglycosides, TEs, nitrobenzene derivatives, and sulfonamide groups. Distribution patterns of the AMR mainly showed AM, PIP, CN, CPD, CFO, CFT, GM, TE, C, SXT (24.64%), followed by AM, PIP, CN, CPD, CFO, CFT GM, TE, and C (13.04%), and AM, PIP, CN, CPD, CFO, CFT GM, ENR, MRB, TE, C, SXT TE, and C (10.14%) (Table-2). Considering all market types, the results showed the highest resistance (100%) to AM, PIP, CN, CPD, CFO, and CFT. Following high resistance rates were GM and TE (>80.0%). Supermarket and pork store isolates showed high resistance to GM, C, and trimethoprim/SXT (>70.00%). However, the isolates from supermarkets did not show resistance to TM and ones from pork stores did not show resistance to FT (Figure-2).

**Table-2:** Antimicrobial resistance patterns in pork from markets in Muang district, Chiang Mai Province.

Multiple drug-resistance patterns	No. of antibiotics	No. of isolation
AM, PIP, CN, CPD, CFO, CFT, GM, TE, C, SXT	10	24.64
AM, PIP, CN, CPD, CFO, CFT GM, TE, C	9	13.04
AM, PIP, CN, CPD, CFO, CFT GM, ENR, MRB, TE, C, SXT, TE, C	14	10.14
AM, PIP, CN, CPD, CFO, CFT, GM, TE, SXT	9	8.67
AM, PIP, CN, CPD, CFO, CFT, GM, ENR, MRB, TE, SXT	11	4.34
AM, PIP, CN, CPD, CFO, CFT, GM, TE	8	4.34
AM, PIP, CN, CPD, CFO, CFT, GM, ENR, MRB, TE, C	11	4.34
AM, PIP, CN, CPD, CFO, CFT, GM, C, SXT	9	4.34
AM, PIP, CN, CPD, CFO, CFT, TE	7	4.34
AM, PIP, CN, CPD, CFO, CFT GM, SXT	8	2.90
AM, PIP, CN, CPD, CFO, CFT, GM	7	2.90
AM, PIP, CN, CPD, CFO, CFT, GM, TM, ENR, MRB, TE, C, SXT	13	2.90
AM, PIP, CN, CPD, CFO, CFT, GM, ENR, MRB, TE, FT, C, SXT	13	1.45
AM, PIP, CN, CPD, CFO, CFT, GM, MRB, TE, FT, C, SXT	12	1.45
AM, PIP, CN, CPD, CFO, CFT, GM, ENR, MRB, TE, FT, C	12	1.45
AM, PIP, CN, CPD, CFO, CFT, ENR, MRB, C, SXT	10	1.45
AM, PIP, CN, CPD, CFO, CFT, GM, TM, TE	9	1.45
AM, PIP, CN, CPD, CFO, CFT, GM, ENR, MRB, C	10	1.45
AM, PIP, CN, CPD, CFO, CFT	6	1.45
AM, PIP, CN, CPD, CFO, CFT, GM, TM	8	1.45
AM, PIP, CN, CPD, CFO, CFT, GM, C	8	1.45

AM=Ampicillin, PIP=Piperacillin, CN=Cefalexin, CPD=Cefpodoxime, CFO=Cefovecin, CFT=Ceftiofur, GM=Gentamycin, TE=Tetracycline, C=Chloramphenicol, SXT=Trimethoprim/sulfamethoxazole, ENR=Enrofloxacin, MRB=Marbofloxacin, TM=Tobramycin, FT=Nitrofurantoin

### Extended-spectrum beta-lactamases-producing *E. coli* genotype

The most common genotypes were *bla*CTX-M groups (100%); *bla*CTX-M-1 (94.02%) and *bla*CTX-M-9 (10.14%), without *bla*CTX-M-2, *bla*CTX-M-8, and *bla*CTX-M-25 followed by *bla*TEM (98.55%). Since the results showed roughly the same proportions of *bla*CTX-M and *bla*TEM genotypes among the three market types, statistical significance was not different (p = 1). In addition, even though the proportion of *bla*CTX-M-9 was smaller than *bla*CTX-M-1, the result did not represent a statistically significant difference between the three markets (p = 0.25 and 0.54, respectively). One isolation from fresh markets was found carrying *bla*SHV (1.45%), without *bla*OXA detection (Table-3).

**Table-3:** Genotype of ESBL-producing *E. coli* in pork from fresh markets (n=35 isolates), pork retail (n=17), and supermarket (n=17).

ESBL-producing *E. coli* genotype	Source						Total		p-value

	Fresh markets		Pork retail stores		Supermarkets				
			
	n	%	n	%	n	%	n	%	
*bla* CTX-M	35	100	17	100	17	100	69	100	1
*bla* CTX-M-1	33	94.29	17	100	15	88.24	65	94.02	0.54
*bla* CTX-M-9	4	11.43	0	0	3	17.65	7	10.14	0.25
*bla* TEM	34	97.14	17	100	17	100	68	98.55	1
*bla* SHV	1	2.86	0	0	0	0	1	1.45	1
*bla* OXA	0	0	0	0	0	0	0	0	NA

*E. coli=Escherichia coli*, ESBL=Extended-spectrum beta-lactamases

### Distribution of genotypic ESBL-producing *E. coli*

The distribution patterns of the ESBL-producing *E. coli* genotypes are shown in Table-4. The coexistence of *bla*CTX-M and *bla*TEM was highly observed (97.10%). *bla*CTX-M, *bla*TEM, and *bla*SHV showed the least common coexistence (1.45%). The frequency of the distribution patterns of *bla*CTX-M groups was high in *bla*CTX-M-1 (89.86%), followed by *bla*CTX-M-9 (5.80%). While, the coexistence of *bla*CTX-M-1 and *bla*CTX-M-9 was 10.15%. The distribution patterns of genotypic ESBL-producing *E. coli* among fresh markets, pork stores, and supermarkets did not show a statistically significant difference (p = 1).

**Table-4:** The distribution patterns of the ESBL-producing *E. coli* genotypes from fresh markets (n=35 isolates), pork retail (n=17), and supermarket (n=17).

Patterns of ESBL-producing *E. coli* genotypes	Source						Total (69)	

	Fresh markets		Pork retail stores		Supermarkets			
			
	n	%	n	%	n	%	n	%
*bla*CTX-M + TEM	33	94.29	17	100	17	100	67	97.10
*bla*CTX-M + TEM + SHV	1	2.86	0	0	0	0	1	1.45
*bla*CTX-M only	1	2.86	0	0	0	0	1	1.45

The distribution patterns of the ESBL-producing *E. coli* genotypes from pork did not show statistically significant difference among fresh markets, pork stores, and supermarkets (p = 1). *E. coli=Escherichia coli*, ESBL=Extended-spectrum beta-lactamases

## Discussion

The study revealed that the proportion of ESBL-producing *E. coli* in pork from markets (69%), which was higher than the previous studies in China (11.76%) [16], United Kingdom (<7%) [14], and Thailand (>50%) [12]. Moreover, the previous studies concerning swine production chain, including pigs, farmers, and the environment in the northern part of Thailand represented more than 50% of ESBL-producing *E. coli* [3, 4]. Foods from animal production are presented as a reservoir of ESBL-producing *E. coli* [22]. The results of the study emphasized the increasing risk of transmission in contaminated food chain and more outbreaks of the resistant pathogen. Extended-spectrum beta-lactamases-producing *E. coli-*positive results were found in supermarkets less than in fresh markets and pork stores due to more strict swine audit process. Suppliers have to be certified by DLD before providing pork products to supermarkets. The assessments include farm systems, slaughterhouse control, pork quality, and retail management [23–26]. In contrast, some shops in fresh markets are not required to obtain any certificates for the particular assessments. Besides, the sales pattern of pork meat in fresh markets basically involves middlemen who directly purchase pork at farms and sell it to vendors in markets, farmers who raise pigs, slaughter it for meat, and sell it on their own. In addition, pork stores supply fresh meat particularly produced by their own farms on their own slaughterhouse evaluation and selling process. This, as a result, can cause non-standardized pork assessment on the ground that consumers prefer buying pork at fresh markets to the other market types. It is evidently due to relatively cheaper prices, easier ways to buy, more accessible locations as well as acquaintanceship with the venders. In other words, pork quality is the secondary reason for most customers [27, 28]. Local people in the north of Thailand, especially, those who prefer the traditional raw pork menu, take relatively high risks of exposure to resistant antibiotics. This can lead to more serious infections associated with the pathogen, more complicated antibiotic treatment, and higher medical expense. Due to the circumstances mentioned, it is important that we, as part of the food chain, take these facts into consideration for a health advantage.

All ESBL-producing *E. coli* were detected as multidrug-resistant. The positive ESBL (100%) were resistant to beta-lactam antibiotic groups, followed by gentamicin which was widely used in animal production in Thailand [3]. Evidently, inappropriate use of antibiotics can lead to a high AMR rate. The resistance to beta-lactam antibiotic groups implies a challenge for treating diseases caused by ESBL-producing *E. coli*. Moreover, it results in critical public health as it is associated with morbidity, mortality, and limited alternatives of treatment. Over 50% of the bacteria with high resistance to TE and C groups were found, even though, interestingly, TE has been restricted since 2003 [29] and C has been prohibited since 2002 [30] in animal production. A previous study in Thailand also reported high levels of TE and C in swine farms, including pigs, farmers, and environment. In addition, coexistence of resistance between antibiotic classes was observed. Multiple drug resistance has become a major problem for clinical therapeutics for antibiotic-resistant genes that can potentially spread through horizontal gene transfer from commensal to environmental species [8]. However, this study showed susceptibility (100%) of the ESBL-producing *E. coli* to IPM. A previous study in China found ESBL-producing *E. coli* (95%) susceptive [16] and another study in Thailand also reported susceptibility (over 98%) of ESBL-producing *E. coli* [19]. Therefore, IPM could be a potential drug choice to cure ESBL-producing *E. coli*-associated infections.

*bla*CTX-M group was predominantly detected in this study, including *bla*CTX-M-1 with the highest rate, followed by *bla*CTX-M-9. Worldwide, previous studies also reported the particular group of genes in humans, animal farms, and foods [3, 17, 19, 31]. However, a study in China found *bla*OXA (28.57%) in retailed fresh pork [16], which was not detected in this study. Evidently, genotypic ESBL-producing *E. coli* commonly vary in different countries and regions because of different antibiotic policies. The coexistence of different genes within the same isolation was noticed; the most common genotypic pattern was the combination of *bla*CTX-M and *bla*TEM. This can lead to enzyme combination issues. Moreover, single isolates with multiple *bla*CTX-M can possibly cause more stubborn infections. Therefore, it is necessary that pork production should be responsibly monitored throughout the process because ESBL-producing *E. coli* are not only disseminated in animals but also in foods, humans, and the environment.

## Conclusion

Pork meat can be prepared in various ways and consumed either cooked or raw. A high percentage of ESBL-producing *E. coli* was found in pork from various market types. In addition, all the isolates were multiple drug-resistant. The predominant genotype was *bla*CTX. Apparently, contaminated pork is one of the transmission routes for the pathogen to food chain. In summary, rational use of antimicrobial drugs, along with choosing pork from reliable sources and hygienic cooking process, are recommended to be practiced with stewardship by every involved unit, from producers to consumers, for the advantage of food safety as well as public health.

## Authors’ Contributions

WS: Data collection, laboratory test, and manuscript. WC: Laboratory test. MI: Data analysis and laboratory test. KNL: Data analysis, prepared figures and tables, and revised manuscript. All authors have read and approved the final manuscript.
